# Hand Replantation With Dorsal Spanning Plate Following a Self-Inflicted Radiocarpal Amputation

**DOI:** 10.7759/cureus.36656

**Published:** 2023-03-24

**Authors:** Wilson C Lai, Cory Pham, Justin P Chan, Brandon E Lung, Gregory H Rafijah

**Affiliations:** 1 Orthopaedic Surgery, University of California Irvine Health, Orange, USA

**Keywords:** upper extremity amputation, radiocarpal amputation, psychiatric, repair, replantation, dorsal spanning plate

## Abstract

Upper extremity amputations represent a prime opportunity to restore function through replantation. There are a variety of options that treating surgeons use to protect neurovascular repairs and restore function including Kirschner wire fixation, external fixation, wrist arthrodesis, and proximal row carpectomy. Additionally, the dorsal spanning plate may be a valuable tool for protecting neurovascular repairs. Compared to temporary immobilization with Kirschner wire fixation, which has previously been described in conjunction with upper extremity replantation, dorsal spanning plates can be left in place for longer durations with a lower risk of loosening and loss of fixation and for preventing postoperative sabotage or repeat amputation of the replant by the patient.

In this article, we describe a unique case of a patient with acute psychiatric illness that presented with a self-inflicted amputation through the radiocarpal joint and was initially treated with emergent replantation and application of a dorsal spanning plate to protect the neurovascular repair from patient sabotage and allow for early rehabilitation. We found the dorsal spanning plate to be an effective option in this complex clinical scenario. This case illustrates the utility of the dorsal spanning plate in protecting complex neurovascular repairs in the setting of severe skeletal and psychiatric instability.

## Introduction

Complete upper extremity amputations are rare but devastating injuries with an estimated prevalence of 11.6 per 100,000 adults [1]. After complete amputation at the wrist joint, successful replantation is critical when possible as there is excellent potential for recovery of function and sensation. Hoang showed that although hand intrinsic muscle function was markedly impaired after replantation, patients recovered satisfactorily for most daily tasks without pain or instability [2].

Bone shortening and stable fixation are critical steps to achieve the final functional recovery, but there is no clear consensus over the best method for bony fixation when performing a replant at the level of the wrist joint. Motion-preserving procedures versus wrist arthrodesis after replantation have all been described; however, others have advocated attempts at joint-preserving procedures to achieve active wrist motion after replantation [3,4]. Typically, expedient bony fixation is done using Kirschner wires or Steinman pins after bone shortening with proximal row carpectomy to protect any neurovascular or tendinous repair. Alternatively, external fixation can be used. However, these methods of fixation do not provide rigid immobilization and precise maintenance of reduction, especially with an unstable radiocarpal joint. The relative stability of wire or external fixation often results in an unstable wrist requiring late arthrodesis. Alternatively, primary wrist fusion takes greater time to perform and deprives the patient of potentially useful future wrist mobility. Furthermore, patients with psychiatric disorders may sabotage their own hand replantation and physically avulse the replanted hand after wire fixation. Dorsal spanning plate fixation may offer an alternative solution to Kirschner wire fixation in patients requiring more stability, especially for those with complicating psychosocial factors.

Although dorsal spanning plate fixation has been described as an effective treatment option for unstable comminuted distal radius fractures with safe and minimal complications, no published studies have described its use specifically in the setting of radiocarpal amputations [5]. Here, we present a unique case study in which a dorsal spanning plate was used after successful replantation from a self-inflicted radiocarpal amputation in a 43-year-old male with acute decompensation of schizophrenic disorder.

## Case presentation

**Figure 1:**
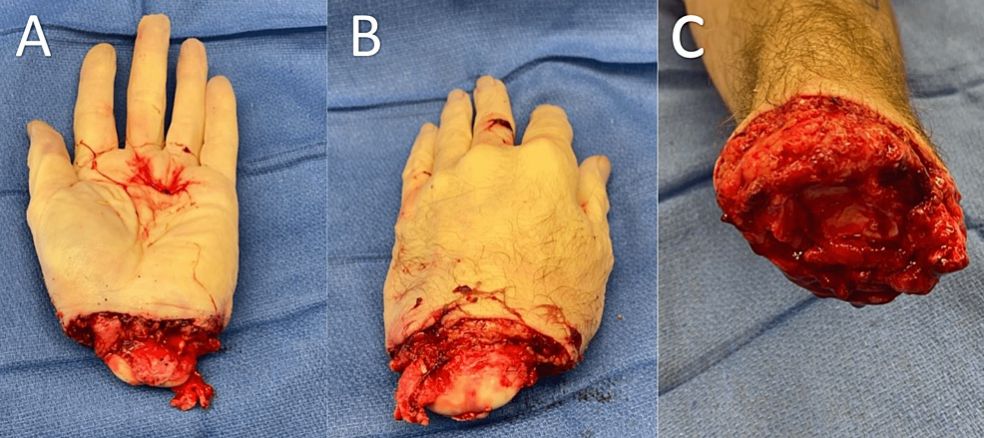
(A-C) Gross specimens showing the amputated hand and amputation stump with exposed lunate and scaphoid fossa on the distal radius

**Figure 2:**
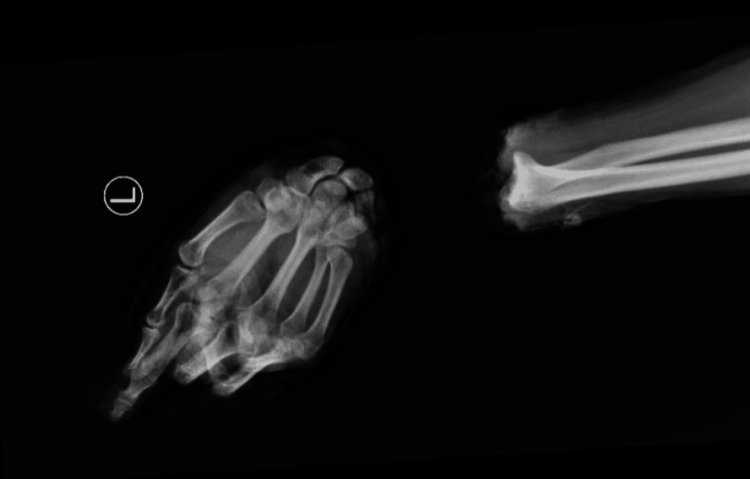
Initial radiographs demonstrating complete amputation through the radiocarpal joint

A 43-year-old right-hand dominant male with a history of schizophrenia and depression presented with a complete amputation at the left radiocarpal joint as a result of an intentionally self-inflicted injury using a sharp knife after reactivation of schizophrenia with the manifestation of mystic delusion. He believed that by amputating his hand at the wrist, he could “donate his hand to a needy family member and that God would help regrow his hand.” The patient stated that it took him 10 minutes to amputate his hand through the left radiocarpal joint, and he denied any pain during the event. Initial injury occurred approximately 45 minutes prior to arrival at our level 1 trauma center with the amputated limb. His amputation was found to be relatively sharp and clean at the level of the radiocarpal joint (Figures 1, 2).

On arrival, he was noted to have an incongruent blunted affect and did not seem to be affected by the self-inflicted trauma. Emergency psychiatry consultation was obtained, and it was determined that the patient was delusional and lacked the capacity to make an informed medical decision. We were able to contact his mother who claimed power of attorney and consented to emergent replantation surgery.

**Figure 3:**
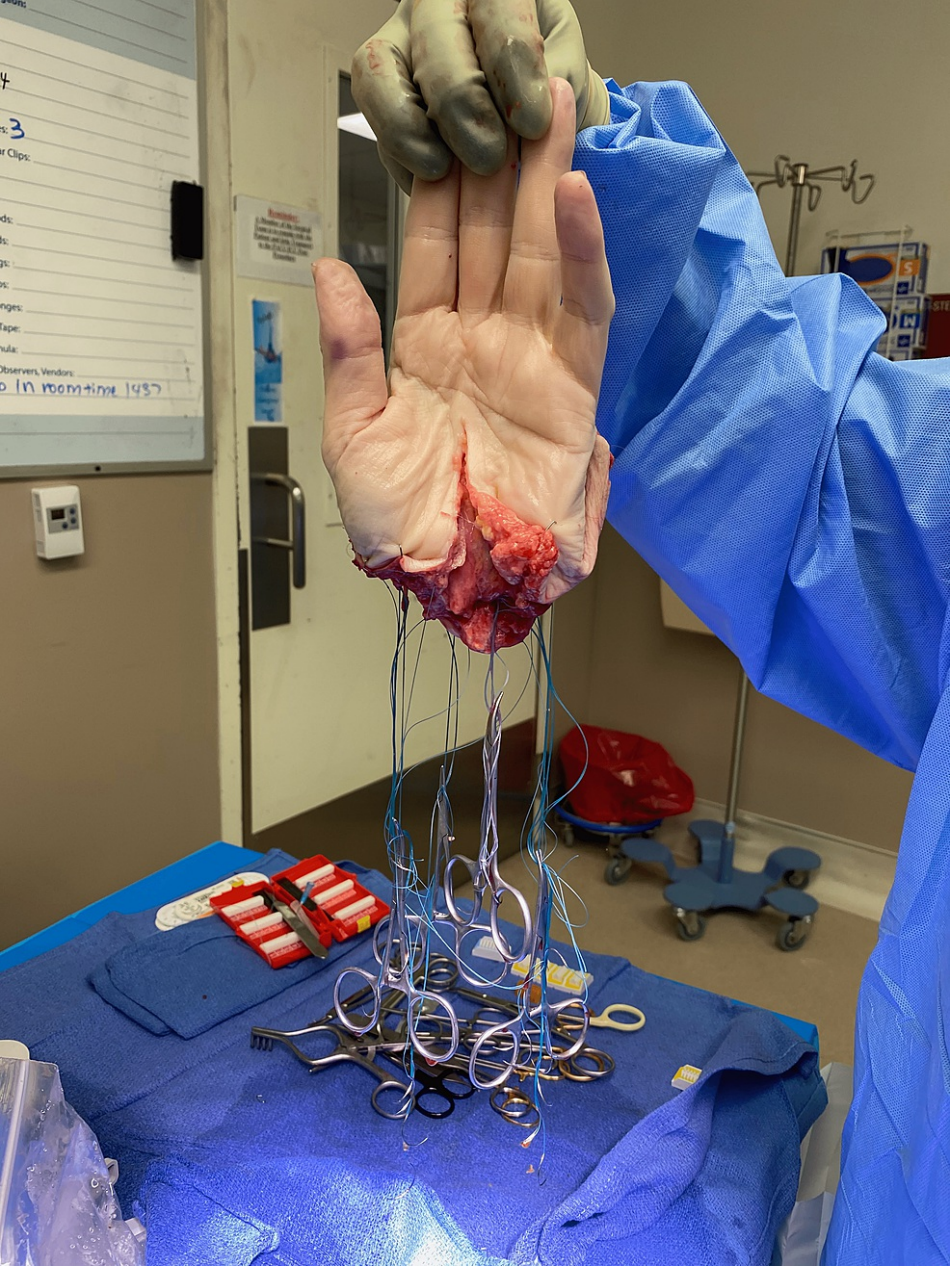
The amputated hand prepared for replantation after the neurovascular structures have been identified and core sutures placed in the extensor and flexor tendons

As a result, the patient underwent emergent replantation of his left upper extremity. To facilitate a tension-free repair of the neurovascular bundle and tendinous structures, bone shortening was provided through proximal row carpectomy before skeletal stabilization. For maximal stability and to prevent sabotage of his replant, skeletal stabilization was first obtained using a dorsal spanning plate with stabilization to the third metacarpal. Suture anchors were placed along the volar and dorsal aspect of the distal radius to help facilitate capsular repair. The extensor tendons were repaired using the modified Kessler technique. Flexor tendons were repaired using the 6-core suture technique with 4-0 FiberLoop sutures (Figure 3).

**Figure 4:**
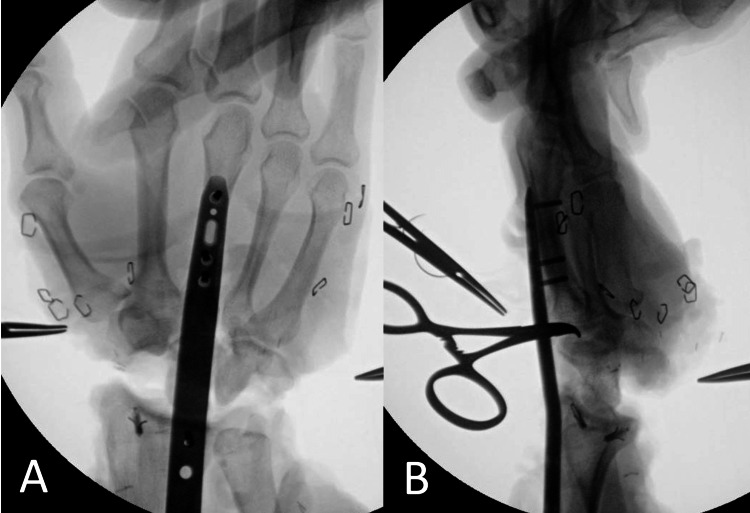
(A, B) Intraoperative fluoroscopic images of the distal aspect of the dorsal spanning plate demonstrating a stable radiocarpal joint status post proximal row carpectomy

Arterial inflow was restored with the ulnar artery repair as it appeared healthier compared to the radial artery, and venous outflow was accomplished by repairing two dorsal veins using vein couplers. The ulnar nerve and median nerve were repaired with 8-0 nylon sutures and fibrin glue, and a nerve protector was applied to further protect our nerve repair. Fluoroscopic images demonstrated stability of our dorsal spanning plate (Figure 4).

**Figure 5:**
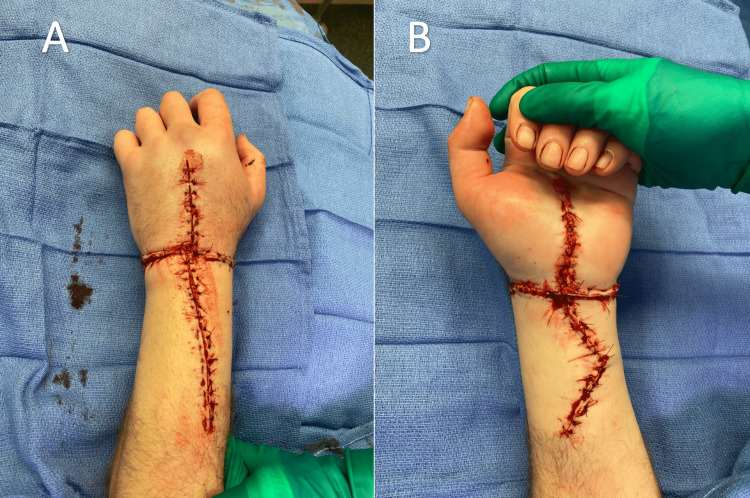
(A, B) Immediate postoperative appearance of the replanted hand without protruding hardware

The wounds were then closed. Total ischemia time was not recorded, and total tourniquet time was 120 minutes. The appearance of the immediately replanted hand is illustrated in Figure 5.

**Figure 6:**
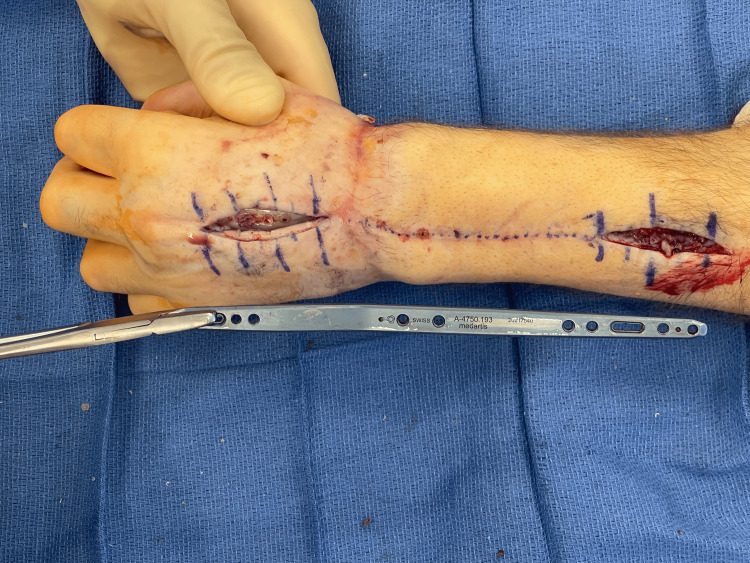
Intraoperative photo demonstrating the minimal incisions required to remove the dorsal spanning plate at 4.5 months after replantation

Postoperatively, the patient was monitored in the surgical intensive care unit (SICU) with consultation from psychiatry. He was diagnosed with acute decompensation of his primary psychotic disorder. Recommendations included Risperdal 2 mg nightly for his psychosis, along with one-to-one monitoring and transfer to an inpatient psychiatric facility when cleared by the hand service. After four months, his wrist was determined to be stable, and we planned for the removal of the dorsal spanning plate. The dorsal spanning plate was removed 4.5 months after the index procedure (Figure 6).

**Figure 7:**
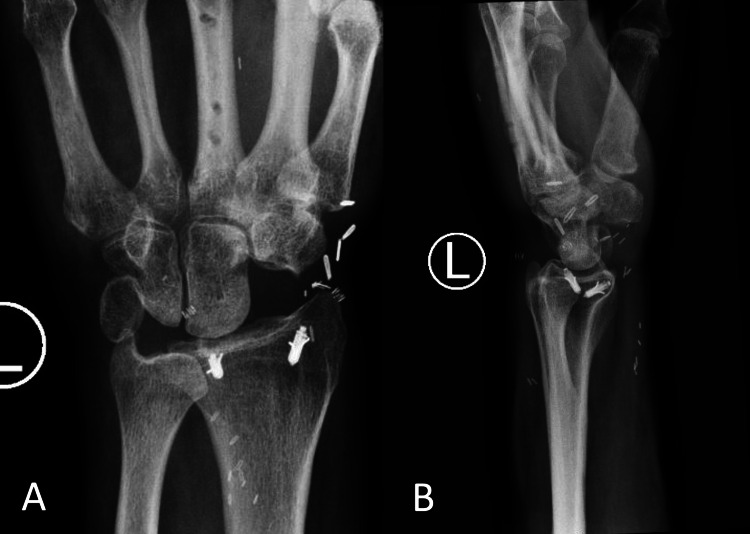
(A, B) Radiographs of the left wrist six months after replantation with proximal row carpectomy and 1.5 months after the removal of the dorsal spanning plate, demonstrating stable alignment

The patient was allowed to return to activity of daily living with weight-bearing to the left upper extremity. He was encouraged to work on a full range of motion of the hand and wrist as tolerated. At six weeks after dorsal spanning plate removal (six months post replantation), the patient was lost to follow-up as he was discharged home and refused clinic follow-up. However, his last radiographs demonstrated a stable radiocarpal joint (Figure 7).

## Discussion

Although rare, complete upper extremity amputations represent a prime opportunity to restore function through replantation. The balance between providing adequate stability to protect the repair and preserving wrist and finger motion remains a challenging problem. Here, we report the use of a dorsal spanning plate in conjunction with a proximal row carpectomy and the replantation of a self-inflicted amputation through the radiocarpal joint. The dorsal spanning plate, a technique that has been proven to be safe and efficacious in the treatment of wrist fractures, may be a valuable tool for protecting neurovascular repairs, preventing self-inflicted sabotage in patients with psychiatric diseases, and allowing early rehabilitation.

Compared to temporary immobilization with Kirschner wire fixation, which has previously been described in conjunction with upper extremity replantation, dorsal spanning plates can be left in place longer with a lower risk of loosening, pin tract infections, loss of fixation, or removal by the patient. It is recognized that dorsal spanning plate application may require additional dissection and time for application over traditional Kirschner wire fixation. However, the enhanced stability provided by the dorsal spanning plate allows for a longer healing period that may improve the success of a motion-sparing procedure such as proximal row carpectomy and prevent the additional morbidity of wrist arthrodesis. Not only does the solid fixation of the dorsal spanning plate prevent self-inflicted sabotage of the replanted hand, but the enhanced stability provided may also permit earlier rehabilitation with potentially enhanced outcomes.

In patients with severe psychiatric illness and demonstrated propensity to cause self-inflicted amputation, the dorsal spanning plate may have the added benefit of making a repeat amputation after replantation more difficult. However, these patients could still cause significant self-harm postoperatively and require closely monitored psychiatric treatment along with a stable support system to obtain long-term success of an upper extremity replant. Furthermore, after the resolution of the patient’s acute psychosis, studies have demonstrated that patients may be quite grateful postoperatively, which further stresses the importance of maintaining stable fixation after hand replantation in this challenging group of patients [6].

## Conclusions

In specific situations requiring prolonged stabilization across the wrist joint to protect a complex soft tissue injury with neurovascular repairs, including replantation, the dorsal spanning plate may be a beneficial option with several unique advantages compared to the existing methods of immobilization. Although not appropriate in every scenario, it represents an additional tool that the hand surgeon can utilize when approaching a challenging repair or major replant, especially in a patient with a psychiatric illness.

## References

[1] Østlie K, Skjeldal OH, Garfelt B, Magnus P (2011). Adult acquired major upper limb amputation in Norway: prevalence, demographic features and amputation specific features. A population-based survey. Disabil Rehabil.

[2] Hoang NT (2006). Hand replantations following complete amputations at the wrist joint: first experiences in Hanoi, Vietnam. J Hand Surg Br.

[3] Woo SH, Lee YK, Lee HH, Park JK, Kim JY, Dhawan V (2009). Hand replantation with proximal row carpectomy. Hand (N Y).

[4] Satria O, Abubakar I, Karda IWM (2019). Replantation at the level of the wrist joint: a case report. J Clin Orthop Trauma.

[5] Lauder A, Agnew S, Bakri K, Allan CH, Hanel DP, Huang JI (2015). Functional outcomes following bridge plate fixation for distal radius fractures. J Hand Surg Am.

[6] Strain JJ, DeMuth GW (1983). Care of the psychotic self-amputee undergoing replantation. Ann Surg.

